# Exploration of serum- and cell culture-derived exosomes from dogs

**DOI:** 10.1186/s12917-018-1509-x

**Published:** 2018-06-08

**Authors:** Matias Aguilera-Rojas, Brit Badewien-Rentzsch, Johanna Plendl, Barbara Kohn, Ralf Einspanier

**Affiliations:** 1 Institute of Veterinary Biochemistry, Department of Veterinary Medicine, Freie Universität Berlin, 14163 Berlin, Germany; 2 Institute of Veterinary Anatomy, Department of Veterinary Medicine, Freie Universität Berlin, 14195 Berlin, Germany; 3 Small Animal Clinic, Department of Veterinary Medicine, Freie Universität Berlin, 14163 Berlin, Germany

**Keywords:** Exosomes, Serum, Cell culture medium, Dog, Transmission electron microscopy, Nanoparticle tracking analysis, Biomarkers

## Abstract

**Background:**

Exosomes are defined as extracellular membrane vesicles, 30–150 nm in diameter, derived from all types of cells. They originate via endocytosis and then they are released through exocytosis to the extracellular space, being found in various biological fluids as well as in cell culture medium. In the last few years, exosomes have gained considerable scientific interest due to their potential use as biomarkers, especially in the field of cancer research. This report describes a method to isolate, quantify and identify serum- and cell culture-derived exosomes from dog samples, using small volumes (100 μL and 1 mL, respectively).

**Results:**

Quantification and sizing of exosomes contained in serum and cell culture samples were assessed by utilizing nanoparticle tracking analysis, transmission electron microscopy and immunoelectron microscopy. Detected particles showed the normal size (30–150 nm) and morphology described for exosomes, as well as presence of the transmembrane protein CD63 known as exosomal marker.

**Conclusions:**

Based on a validated rapid isolation procedure of nanoparticles from small volumes of different types of dog samples, a characterization and exploration of intact exosomes, as well as facilitation for their analysis in downstream applications was introduced.

## Background

Exosomes are extracellular nano-sized membrane vesicles, reported as 30–150 nm in diameter, derived from all types of cells and released into practically all biological fluids such as blood, urine, cerebrospinal fluid, milk, sputum, saliva, seminal fluid, as well as into cell culture medium [1, 2]. These vesicles originate via endocytosis, initially forming endosomes and followed by invagination of the endosomal membrane to create multivesicular bodies (MVBs). Afterwards through exocytosis, the content of the MVBs is released as exosomes to the extracellular space once merging with the plasma membrane [3, 4].

The exosomal membrane consists mostly of lipids and proteins, while the luminal cargo is mainly represented by proteins and nucleic acids, including mRNAs, microRNAs, other non-coding RNAs and DNA [5–7]. Exosomes have been proven to possess several functions, for instance, intercellular communication, genetic exchange and antigen presentation, allowing cells to transport their cargo in a short and long distance manner and subsequently having a significant effect at a cellular and biological level [6, 7]. Since exosomes are of endosomal origin, they contain a distinct set of proteins involved in membrane transport and fusion (e.g. Rab GTPases, annexins, flotillin), biogenesis of MVBs (Alix, TSG101), major histocompatibility complex class I and II, in processes requiring heat shock proteins (hsc70 and 90), integrins and tetraspanins (e.g. CD63, CD9, CD81 and CD82) [6, 8, 9]. Even though some of these proteins are used as exosome markers, exosomal protein composition might differ based on the origin of the cells or tissue [7, 10].

Analyses of cargo proteins and nucleic acids present in exosomes show significant potential to be employed as exosomal biomarkers. Taking this into consideration, together with the ability to easily isolate exosomes from body fluids (liquid biopsy), these vesicles may deliver an additional valuable non-invasive biomarker for predisposition, prognosis and treatment monitoring in the cancer research field [7, 11]. Furthermore, when understanding endogenous transmission of distinct macromolecules between tissues via exosomes, a (dys) functional cell-cell communication could be focused (diagnostic tool) and subsequently modified (therapeutic tool).

In this report we describe a method to isolate and identify serum- and cell culture-derived exosomes from dog samples. This study provides comprehensive techniques such as transmission electron microscopy, nanoparticle tracking analysis and immunodetection to identify and characterize exosomes, allowing them to be quantified and sized, as well as characterized through specific morphology and a distinct protein expression.

## Methods

### Blood serum

Samples (*n* = 10) were gathered from 5 female and 5 male dogs of different ages (between 1 and 7 years old), non-cancer (*n* = 6) and cancer patients (*n* = 4), presented at the Small Animal Clinic, Department of Veterinary Medicine at the Freie Universität Berlin. Blood samples were collected in tubes without anticoagulant and left at room temperature to allow clotting for 30 min to 2 h. The main portion of the serum was used for the original diagnostic laboratory analyses, while the remaining amount was employed for this study. The protocol to separate and store serum was based on a published technical note from QIAGEN (miRNeasy Serum/Plasma Handbook 02/2012). Briefly, tubes were first centrifuged at 2000 x g for 10 min at 4 °C to separate residual cellular components of the blood. The supernatant was then placed in another tube and centrifuged at 16,000 x g for 10 min at 4 °C to separate any left cellular debris. Afterwards, the purified serum was taken and stored in − 80 °C until exosome isolation.

### Cell cultures

#### C2 cell line

C2 cells, a canine mast cell tumour cell line, were kindly provided in August 2016 by Dr. Patrice Dubreuil (Centre de Recherche en Cancérologie de Marseille, Inserm U1068, Marseille, France), after previous consent of the cell line originator, Dr. Warren Gold (University of California San Francisco, School of Medicine, California, USA) [12]. Cells were cultured in RPMI 1640 medium, supplemented with 10% foetal bovine serum (FBS) superior, 100 U/mL penicillin/streptomycin (all from Biochrom, Berlin, Germany), 1 mM/mL sodium pyruvate and 2 mM/mL glutamine (both from Sigma, MO, USA), and incubated in a 5% CO_2_ atmosphere at 37 °C. Special culture conditions were applied before exosomes were harvested (see exosome isolation paragraph).

#### Primary canine fibroblasts culture

Fibroblasts (FBs) were obtained from a portion of healthy skin of a female Golden Retriever, within 20 min after the animal was euthanized at the Small Animal Clinic, Department of Veterinary Medicine at the Freie Universität Berlin. The skin was collected in sterile Dulbecco’s phosphate buffer saline (DPBS) (Sigma, MO, USA) and then placed in a Petri dish. Dermis was separated from epidermis using sterile forceps and scalpels. The dermis was cut in small pieces (1 × 3 mm approx.) and washed in DPBS, supplemented with 100 U/mL penicillin/streptomycin and 250 μg/mL amphotericin B (Biochrom, Berlin, Germany). Then, a 5 min centrifugation at 300 x g was performed and the supernatant was discarded. The sediment, representing the FBs, was resuspended in an enzymatic digestion medium containing 0.15% collagenase I (Biochrom, Berlin, Germany), RPMI 1640 medium, supplemented with antibiotic and fungicide as described above, and 1% 70 mM CaCl_2_ (Merck, Darmstadt, Germany). The sample was transferred into a Petri dish and incubated at 37 °C for 2 h under constant agitation, then placed into a sterile 50 mL tube and centrifuged 5 min at 300 x g, the supernatant was discarded. The pellet was washed twice in warm (37 °C) RPMI 1640 medium, supplemented with 20% FBS, 100 U/mL penicillin/streptomycin, 250 μg/mL amphotericin B, 1 mM/mL sodium pyruvate and 2 mM/mL glutamine, and centrifuged 5 min at 300 x g. Lastly, the resulting pellet was seeded in a T25 flask in 7 ml of the same medium used for the last two washing steps and incubated in a 5% CO_2_ atmosphere at 37 °C. The first passage was performed 10 days after seeding and passage number 5 was used for the exosome isolation. Special culture conditions were applied before exosomes were harvested (see exosome isolation paragraph).

### Exosome isolation

#### Serum samples

Exosome isolation from serum samples was accomplished using a commercial kit (Total Exosome Isolation Reagent – from serum; Invitrogen, Vilnius, Lithuania) following the manufacturer’s protocol. Briefly, purified serum was passed through a 0.22 μm pore PVDF filter (Rotilabo, Karlsruhe, Germany). After that, 100 μL of filtered serum was mixed with 20 μL of reagent and incubated at 4 °C for 30 min. Then, samples were centrifuged at 16,000 x g for 10 min at room temperature and the supernatant was discarded. The pellet containing exosomes was resuspended in 20 to 50 μL of DPBS, depending on the downstream applications.

#### Cell culture samples

In cell culture medium from the C2 cell line and from the primary FBs culture, exosome isolation was performed utilizing a commercial kit (Total Exosome Isolation Reagent – from cell culture media; Invitrogen, Vilnius, Lithuania), although some modifications to the manufacturer’s protocol were applied. For this purpose, prior to culturing cells for exosome isolation, 50–80% confluent C2 cells and primary FBs were washed twice in DPBS and further cultured in an exosome-free medium as described above, except for using exosome-depleted FBS (Gibco, USA). Briefly, cell culture medium was harvested after 48 and 72 h of incubation with exosome-depleted medium and centrifuged at room temperature; first, 5 min at 300 x g to remove floating cells and a subsequent 30 min 3000 x g centrifugation to eliminate cellular debris. Afterwards, the purified medium was passed through a 0.22 μm pore PVDF filter and then 1 mL of filtered medium was mixed with the volume of reagent indicated by the manufacturer. The mixture was incubated at 4 °C overnight and finally centrifuged at 4 °C at 11,000 x g for 60 min. The pellet containing exosomes was re-suspended in 20 to 50 μL of DPBS, depending on the downstream applications.

### Transmission electron microscopy (TEM)

To identify exosomes and investigate their ultrastructural morphology, a Zeiss EM 109 transmission electron microscope (Carl Zeiss, Oberkochen, Germany) operating at 80 kV was utilized, following the protocol developed by Théry et al. (2006) [13], with some modifications.

#### Native exosomes

For analysis of native exosomes, PBS-suspensions containing unfixed exosomes were differentially diluted in filtered PBS (0.22 μm pore PVDF filter). For serum-derived exosomes; undiluted suspension and 1:100, 1:1000 and 1:2000 dilutions were used, while for culture-derived exosomes; undiluted suspension and 1:50, 1:100 and 1:200 dilutions were applied. Formvar-carbon-coated 100 mesh nickel grids (Plano, Wetzlar, Germany) were laid on a 5 μL drop of the exosome-suspension and incubated 20 min at room temperature for adhesion (coated side of the grid facing the suspension), then washed 3 times for 3 min in filtered PBS. Next, grids were placed 2 times on drops of 50 mM glycine/PBS for 3 min and then transferred to a drop of 0.5% bovine serum albumin (BSA)/PBS blocking solution for 10 min. For contrasting the exosomes, grids were laid on 2% uranyl acetate drops for 6 min, followed by 2 washes with distilled water. Grids were allowed to dry overnight.

#### Immuno-gold labelled exosomes

For examination of immuno-gold labelled unfixed exosomes, anti-CD63 (ABIN1440014, antibodies-online), a goat polyclonal multi-species primary antibody, and a secondary antibody anti-goat IgG (whole molecule) labelled with 10 nm gold (Sigma, MO, USA) were used. The first part of the protocol was identical to the procedure for analysis of native exosomes up to placing the grids on drops of 0.5% BSA/PBS blocking solution for 10 min. This was followed by an incubation step for 2 h at room temperature with the primary antibody anti-CD63 (dilution 1:50 in 0.5% BSA/PBS). Afterwards, grids were washed 5 times for 3 min in drops of 0.5% BSA/PBS and an additional incubation with the secondary antibody anti-goat IgG-10 nm gold (dilution 1:50 in 0.5% BSA/PBS) was performed for 1 h at room temperature. Grids were washed again 5 times for 3 min in 0.5% BSA/PBS drops, and then laid on 2% uranyl acetate drops for 6 min for contrasting, followed by 2 washes in distilled water. Grids were allowed to dry overnight.

### Nanoparticle tracking analysis (NTA)

Quantification and size determination of dog exosomes purified from serum and cell culture medium was assessed by using the NanoSight NS500 instrument (Malvern, Worcestershire, UK). The NTA 3.0 (build 0064) software visualizes and analyses nanoparticles in real time by associating Brownian motion with particle size. Fresh serum- and cell culture-derived exosomes samples were processed in duplicate and diluted in filtered PBS (0.22 μm pore PVDF filter) until reaching a concentration between 10 and 100 particles per image (optimal ~ 50 particles per image) before examination with the NTA system [14]. The instrument was set up to operate at 25 °C, three videos, 30 s each, were recorded for each specimen and outcomes were analysed with the NTA software.

### Statistical analysis

The data analysis was performed using the software Microsoft Excel 2010 (Microsoft, Redmond, WA, USA), through one-way analysis of variance (ANOVA) and Bonferroni corrected post-hoc Student’s t-tests. *P* value < 0.05 was considered as significant.

## Results

A rapid protocol was validated to isolate nanoparticles from dog samples suitable to further detect size, quantity and evaluate selected protein expression.

### Size and quantification of exosomes by NTA

**Fig. 1:**
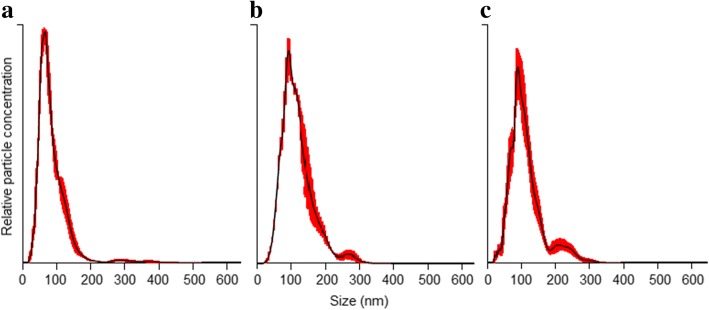
Nano track analysis size distribution of exosomes isolated from samples of canine origin. **a** blood serum-derived exosomes, **b** C2 cell line culture-derived exosomes and (**c**) primary fibroblasts culture-derived exosomes. Red error bars indicate +/− standard error of the mean

A suitable real-time visualization and analysis of exosomes present in fluid samples could be easily performed by the NTA system, both in blood serum (Table 1, Fig. 1a) and in culture media (Table 2, Fig. 1b and c).

**Table 1:** Exosome concentration and size distribution

Sample ID	Exosome concentration (xE10/mL)	Particle size mean (nm)	Type of sample
S1	403.2 +/− 25.8	71.3 +/− 3.1	Non-cancer
S2	107.4 +/− 6.8	90.5 +/− 14.5	Non-cancer
S3	322.8 +/− 24.0	89.5 +/− 1.4	Non-cancer
S10	198.0 +/− 19.9	89.9 +/− 13.2	Non-cancer
SHB	374.4 +/− 21.8	112.5 +/− 12.8	Non-cancer
SNT	219.6 +/− 17.3	111.3 +/− 10.7	Non-cancer
S8	397.2 +/− 18.6	113.9 +/− 9.0	Splenic mast cell tumour
SP	225.6 +/− 10.4	99.0 +/− 8.4	Prostatic carcinoma
S15	500.4 +/− 76.4	84.7 +/− 1.5	Perianal adenoma
SVT	277.2 +/− 11.3	84.5 +/− 0.6	Vaginal leiomyosarcoma

Serum-derived exosomes from non-cancer and cancer dog patients (mean +/− standard error)

**Table 2:** Concentration and size distribution of exosomes

Sample ID	Exosome concentration (xE10/mL)	Particle size mean (nm)	Type of sample
C248	17.5 +/− 0.9	120.9 +/− 2.1	C2 cells culture medium, 48 h incubation
C272	12.8 +/− 1.7	118.0 +/− 4.5	C2 cells culture medium, 72 h incubation
FB48	6.4 +/− 0.7	110.3 +/− 5.0	Primary FBs culture medium, 48 h incubation
FB72	7.3 +/− 1.5	129.0 +/− 7.4	Primary FBs culture medium, 72 h incubation

Culture medium-derived exosomes from C2 cell line and primary fibroblasts, after 48 and 72 h of incubation under exosome-free media conditions (mean +/− standard error)

#### Serum samples

100 μL of canine serum was employed to isolate serum-derived exosomes from 6 non-cancer and 4 cancer dog patients. Most of the observed nanoparticles were found to be 30 to 150 nm in diameter (Table 1, Fig. 1a), i.e. the normal size described for exosomes [1, 2], however few particles showed a larger size. The mean size range for serum-derived exosomes observed was between 71.3 +/− 3.1 and 113.9 +/− 9.0 nm. In terms of exosome concentration, in non-cancer patients nanoparticle concentration (xE10/mL) was between 107.4 +/− 6.8 and 403.2 +/− 25.8, while in cancer patients the lowest and the highest concentrations (xE10/mL) were 225.6 +/− 10.4 and 500.4 +/− 76.4, respectively. Nevertheless, no significant difference (*P* > 0.05) between non-cancer and cancer samples could be calculated, although a large variation within individual samples was detected.

#### Cell culture medium samples

Exosomes could be isolated from 1 mL of culture medium obtained from cultured C2 cells and primary FBs, after 48 and 72 h of incubation under exosome-depleted media conditions. Likewise in the analysis of dog serum samples, the commercial kit was found suitable for isolating exosomes derived from dog cell cultures. The majority of the nanoparticles exhibited the normal size described for exosomes, 30 to 150 nm in diameter (Table 2, Fig. 1b and c) [1, 2], while a small number was found to be in the 150–300 nm range. The mean size range for culture-derived exosomes observed in both types of cultures and both time-points was between 110.3 +/− 5.0 and 129.0 +/− 7.4 nm. In terms of nanoparticle quantification, after 48 and 72 h C2 cells cultures showed a significant (*P* < 0.05) 2–3-fold higher exosome concentration compared to primary FBs cultures but no difference (*P* > 0.05) between incubation times (48 vs. 72 h) was observed in either group.

#### Negative controls

To screen for potentially contaminating particles, samples from all solutions used (PBS, RPMI 1640 medium, RPMI 1640 + exosome isolation kit, and complete culture medium + exosome isolation kit) were analysed as negative controls. The number of particles detected in these fluids was low and did not affect the total concentration of exosomes per mL counted by the NTA system. Therefore, exogenous contamination interacting with a valid characterization of canine exosomes can be excluded as a factor in our system (Table 3).

**Table 3:** Negative controls

Sample ID	Exosome concentration (xE7/mL)	Particle size mean (nm)	Type of sample
CN1	0.88 +/− 0.36	188.2 +/− 62.1	PBS
CN2	1.33 +/− 0.75	138.9 +/− 69.6	RPMI 1640
CN3	1.40 +/− 0.34	130.4 +/− 11.5	RPMI 1640 + Exosome isolation kit
CN4	1.26 +/− 0.61	147.6 +/− 22.6	Complete culture medium + Exosome isolation kit

Solutions employed during harvest and dilution processing of exosomes (mean +/− standard error)

### Exosome morphology by TEM

**Fig. 2:**
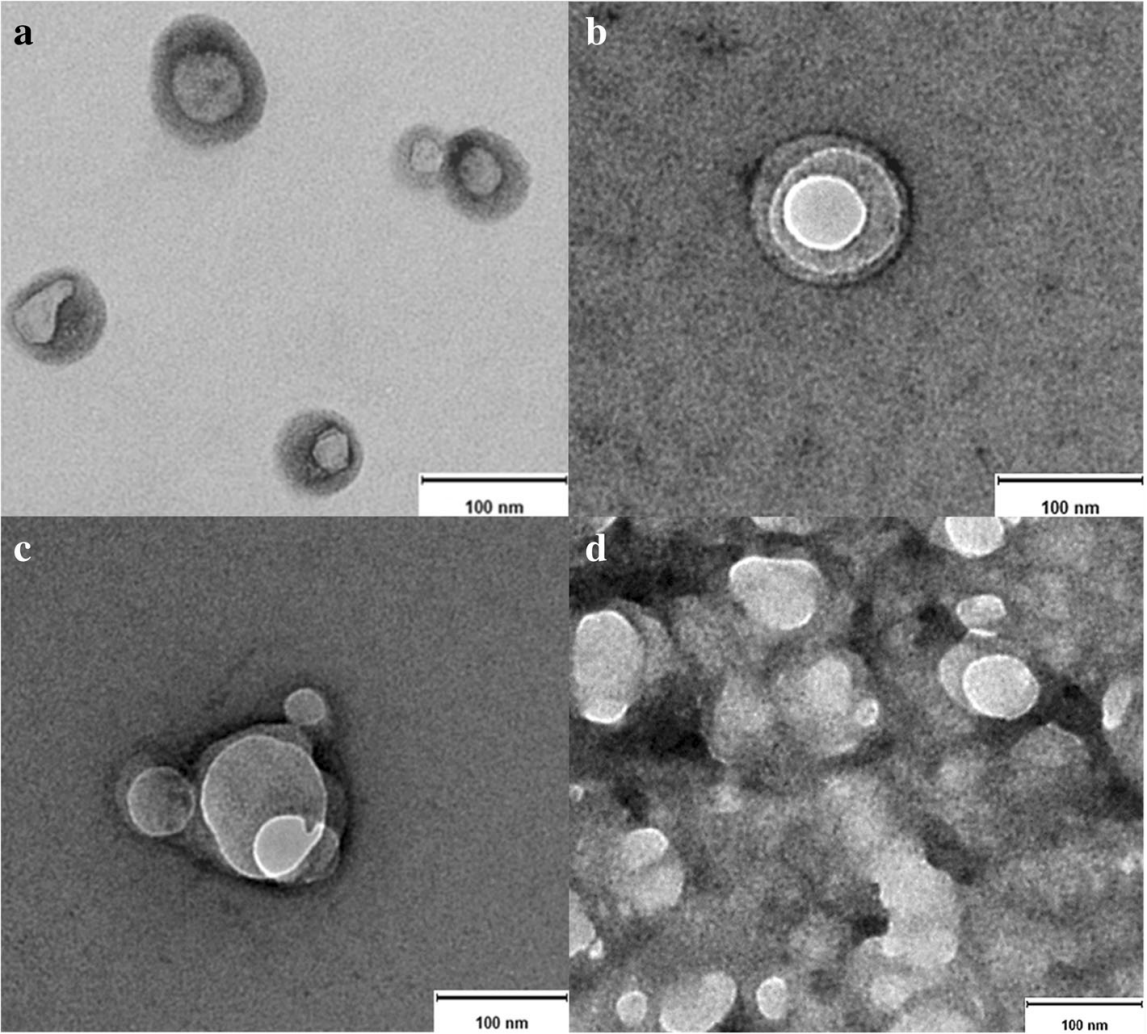
Transmission electron microscopy of native exosomes isolated from samples of canine origin. **a** blood serum-derived exosomes, **b** C2 cell line culture-derived exosomes and (**c** and **d**) primary fibroblasts culture-derived exosomes. Size bar = 100 nm

General morphology and ultrastructure of serum- and culture-derived exosomes of canine origin was assessed by using TEM technology, allowing visualization of the characteristic central depression or “cup shape” of exosomes [15, 16], either single (Fig. 2a and b) or aggregated (Fig. 2c and d). All samples revealed single and aggregated nanoparticles; non-diluted samples showed a higher number of exosome aggregates, whereas samples diluted 1:2000 displayed more individual exosomes, yet it was more difficult to localize them on the grids. Morphology and size of the depicted nanoparticles correspond to their exosomal origin, as described in several studies performed in samples of human fluids and cell culture origin [1, 13–15].

### Protein expression by immunoelectron microscopy

**Fig. 3:**
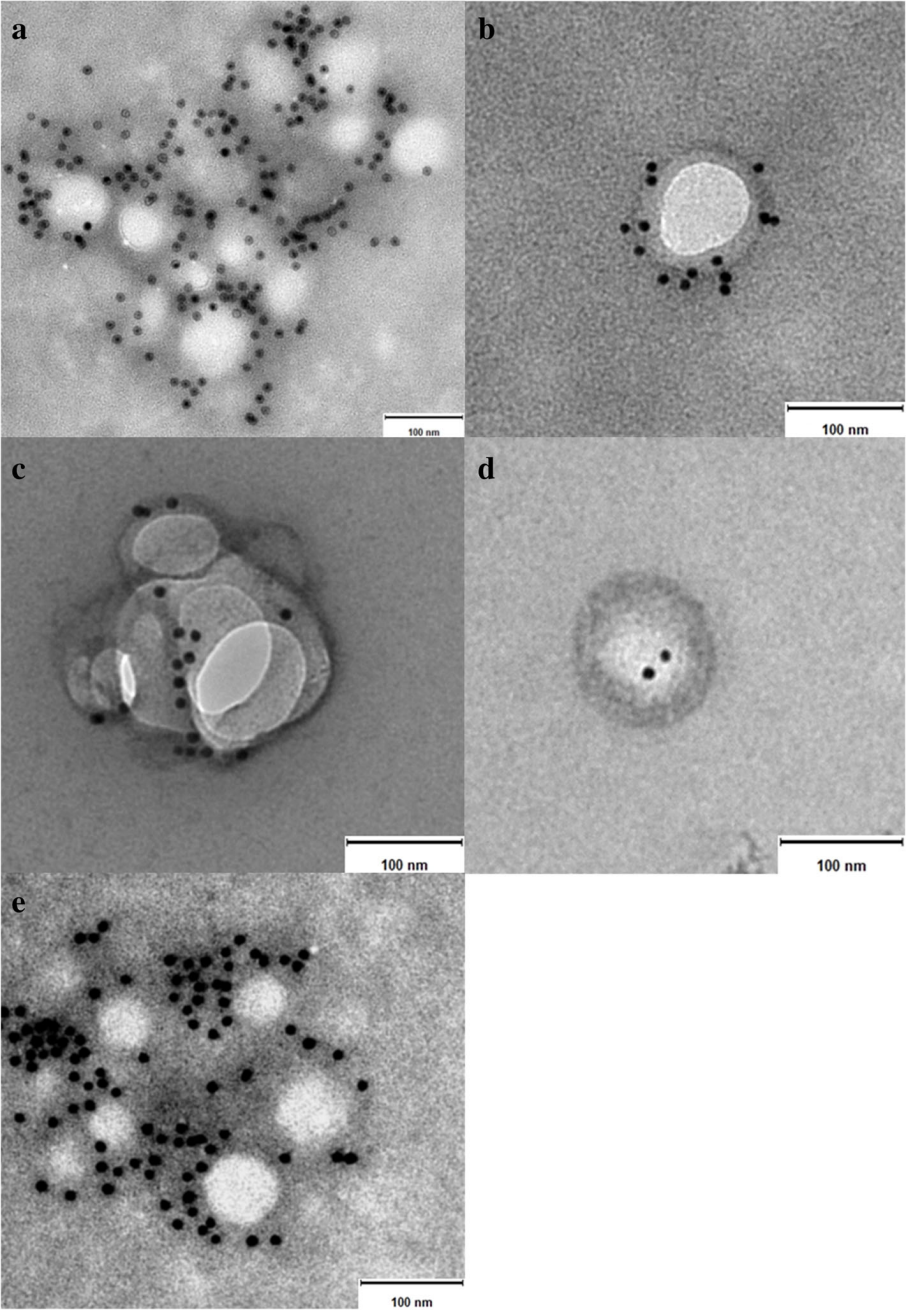
Immunoelectron microscopy images of exosomes isolated from samples of canine origin. **a** and **b** serum-derived exosomes, **c** C2 cell line culture-derived exosomes, **d** and **e** primary fibroblasts culture-derived exosomes. Note the gold particles bound to the exosome membrane indicating presence of the tetraspanin CD63. Size bar = 100 nm

Results presented in Fig. 3 revealed the presence of the transmembrane protein CD63 in all samples investigated in this study. It is important to note that not every single exosome observed by TEM expressed this protein. Indeed, the number of exosomes negative for CD63 was slightly greater to the number of exosomes expressing the protein.

## Discussion

### Size and quantification

The commercial kit used states a simple and quick precipitation method for isolation of intact exosomes, allowing them to be collected by a short, low-speed centrifugation easily applicable in most clinical laboratories [2]. The introduced NTA technology overcomes some limitations inherent to TEM-based methods, such as lack of absolute quantification and quick size determination of exosomes, as well as time-consuming protocols for sample preparation. For that reason, NTA-based procedures appear highly suitable to rapidly characterize size distribution and number of exosomes. However, the NTA system is not able to distinguish between extracellular vesicles (EVs) and other similar sized particles, such as clusters of exosomes, cellular debris or protein aggregates. Moreover, especially when working with precipitation methods, co-isolation of non-exosomal particles, for instance larger serum/plasma protein aggregates or lipoproteins, cannot be excluded [1, 17, 18]. These data might explain why we were able to also observe minor signals showing particles between 150 and 400 nm in the size distribution graph (Fig. 1) in addition to the major peak around 100 nm.

In our approach, culture-derived exosomes were found to have a significantly larger average size than serum-derived specimens, but an obvious size variation between both types of cell cultures was not found. Different studies have provided evidence that EVs vary in size depending on their cells of origin and there are even data published showing variation based on the method of visualization [19–21].

The exosome quantification variability between both cell culture types under the same culture conditions might be explained by the fact that C2 cells represent a cancer cell line. Since exosome secretion is normally increased in cancer [22], a higher exosome concentration in C2 cell medium was expected. Compared to primary FBs, the faster growth rate of C2 cells determines the number of cells contained in each culture flask, an aspect that certainly influences the exosome production. Moreover, it has been well documented that some types of cancer cell lines shed higher amounts of exosomes than others and conditions like hypoxia may increase exosome production up to 90% [23, 24]. Many other elements can also affect exosome shedding in normal and diseased cells, including chemical factors like, calcium, calcium ionophores, phosphatidylinositol 3-kinase, and pH, as well as physical factors such as heat, ischemia, cellular stresses, and loss of cellular attachment [25].

Compared to serum samples, the number of exosomes found in cell culture media was significantly lower. It is known that in vivo exosomes are shed by all types of cells, in normal and diseased conditions [1, 2]. Taking that into consideration, along with the intercellular cross-talks occurring in complex organisms, the total number of cells in a living organism (a dog in this case) releasing exosomes into all body-fluid compartments, is in fact not comparable to the limited number of cells (2–3 × 10^6^) contained in our in vitro culture system.

It is worth to mention that although an aim of this report was to isolate and identify exosomes from different dog serum samples, no differences in size distribution and quantification between non-cancer and cancer dog patients were noticed. Hence, further investigations exploring potential variations between healthy and diseased groups including a larger number of individuals shall follow, since some reports have already shown that cancer cells secrete more exosomes than non-cancer cells [22].

### Morphology and protein expression

Electron microscopy allowed the assessment of morphology and protein expression. Since most optical methods using light scattering to analyse substances or matter, such as flow cytometry and optical microscopy, are hardly able to detect particles smaller than 200 nm, TEM is essential to study the morphology of exosomes and is considered the standard method in this regard [15, 26]. When referring to morphology of nanoparticles, it involves their overall shape, while TEM detects also ultrastructural differences in their shape, contrast and surface patterns [3]. Although we and other researchers described the morphology of exosomes as cup shaped when observed by TEM, it seems to be an artefact generated by fixation and/or contrasting steps [13, 15], that is also associated with shrinking of vesicles [27, 28]. Studies employing scanner electron microscopy and cryo-electron microscopy revealed that exosomes have indeed a round/spherical shape [15, 20, 29, 30].

Immunoelectron microscopy allowed the detection and direct imaging of the transmembrane protein CD63, which bound to a selective secondary antibody labelled with gold particles [31] (Fig. 3). Exosomes represent a heterogeneous population of EVs expressing diverse patterns of molecules. Numerous studies have shown that some of these molecules are found frequently in exosomes, and therefore, they have gained support to be used as exosomal markers, e.g. proteins [13, 15, 31]. Since they all bear an endosomal origin, it is expected that exosomes contain different cargos of tetraspanin proteins, a family of membrane proteins. The tetraspanin CD63 is currently being used widely as a molecular exosome marker by diverse research studies in this field [7, 10]. The tetraspanin family includes a large amount of transmembrane proteins and only the most common members are made available as molecular exosomal markers, including CD63, CD9, CD81 and CD82 [5, 7, 10]. Several investigations have already demonstrated that the molecular characteristics vary broadly among exosomes from different sources, even across exosomes secreted by the same type of cells [10, 22, 25]. Accordingly, the fact that not all of the observed exosomes expressed CD63 was indeed contemplated.

## Conclusion

Our results evidence the feasibility to easily and rapidly isolate intact exosomes from small volumes of serum, as well as from a tumour cell line and a primary fibroblast culture, all from dog origin, allowing nanoparticles to be analysed in downstream applications. The NTA system provides a quick and easy way to size and quantify exosomes, while TEM facilitates the morphology assessment and distinct immunodetection. The exosome research field has in the past years become an emerging area among researchers of all biological sciences. However, in veterinary medicine it is not yet a well-developed matter. Hence, by demonstrating techniques of isolation, characterization and exploration, this report supports the data until now available in the veterinary diagnostic field, encouraging scientists and clinicians to further explore exosomes of canine origin.

## Abbreviations

ANOVA: Analysis of variance
BSA: Bovine serum albumin
DPBS: Dulbecco’s phosphate buffer saline
EVs: Extracellular vesicles
FBs: Fibroblasts
FBS: Foetal bovine serum
MVBs: Multivesicular bodies
NTA: Nanoparticle tracking analysis
PBS: Phosphate buffer saline
PVDF: Polyvinylidene fluoride
TEM: Transmission electron microscopy
